# Follicular fluid SCUBE1 as a novel angiogenesis-associated biomarker in women undergoing ART: a pilot study reflecting the follicular vascular microenvironment

**DOI:** 10.1007/s10815-026-03811-7

**Published:** 2026-01-27

**Authors:** Yusuf Ziya Kizildemir, Hacer Uyanikoglu, Mehmet Incebiyik, Helin Kalir, Sezin Eda Karsli, Işil Işik Okuyan, Bekir Kahveci, Merve Civelek, Cagri Kutlugun Emral

**Affiliations:** 1 Department of Obstetrics and Gynecology, Harran University, Sanliurfa, Turkey; 2 Department of Nursing, Institute of Health Sciences, Harran University, Sanliurfa, Turkey; 3 Department of Obstetrics and Gynecology, Sanliurfa Training and Research Hospital, Sanliurfa, Turkey

**Keywords:** SCUBE1, Follicular fluid, Angiogenesis, Ovarian response, IVF

## Abstract

**Objective:**

To evaluate follicular fluid (FF) SCUBE1 levels as a novel angiogenesis-associated biomarker and investigate its role in reflecting the follicular vascular microenvironment and predicting ovarian response in women undergoing assisted reproductive technology (ART).

**Methods:**

This prospective pilot study included 63 women undergoing IVF/ICSI. Paired serum and FF samples were analyzed. Patients were stratified into Poor (< 4 oocytes), Normal (4–10), and High (> 10) responders. SCUBE1 levels were measured via ELISA. The study primarily investigated the relationship between intrafollicular SCUBE1 and the magnitude of ovarian response as an indicator of the follicular “angiogenic switch.”

**Results:**

Serum SCUBE1 levels decreased significantly during ovarian stimulation (*p* < 0.001). However, FF SCUBE1 levels were approximately twofold higher in High Responders compared to Poor Responders (174.04 ± 112.45 vs. 84.66 ± 73.15 ng/mL, *p* = 0.043). A significant upward trend was confirmed across responder categories (Jonckheere-Terpstra, *p* = 0.032). In ROC analysis, FF SCUBE1 demonstrated promising predictive value for high ovarian response (AUC = 0.718, 95% CI: 0.532–0.904, *p* = 0.048). Notably, a cut-off of 59.13 ng/mL yielded a clinically useful 100% Negative Predictive Value (NPV). SCUBE1 levels did not correlate with oocyte maturation, fertilization, or clinical pregnancy.

**Conclusion:**

Follicular fluid SCUBE1 is a dynamic marker of the follicular vascular microenvironment rather than a direct indicator of oocyte genetic competence. Elevated levels in high responders reflect the intensified angiogenic support required for multiple follicle development. Low FF SCUBE1 may serve as a clinical “red flag” for compromised follicular vascularization in cases of unexpected poor response.

## Introduction

Infertility represents a global health challenge affecting approximately 15% of reproductive-aged couples, carrying profound psychosocial implications alongside its physiological burden [1]. In Assisted Reproductive Technologies (ART), clinical success is fundamentally limited by the *quantity* and *quality* of retrieved oocytes [2]. Although markers such as Anti-Müllerian Hormone (AMH) and Antral Follicle Count (AFC) are currently accepted as the gold standards for the quantitative assessment of ovarian reserve, they remain insufficient in predicting the developmental potential of the oocyte or the quality of the follicular microenvironment. Consequently, there is a pressing need for novel, follicle-specific biomarkers capable of more precisely reflecting oocyte quality and anticipating ART outcomes, particularly implantation and pregnancy success [3].

Oocyte maturation occurs within the follicular fluid (FF), a compartment that facilitates bidirectional communication and metabolic exchange between granulosa cells and the oocyte. This dynamic microenvironment is rich in steroid hormones, growth factors, cytokines, and various metabolites [4]. Folliculogenesis is characterized by rapid tissue proliferation and remodeling, necessitating intense angiogenesis and increased vascular permeability to meet the metabolic demands of the developing oocyte [5]. Establishing a dense vascular network within the theca layer is vital for ensuring gonadotropin delivery and maintaining intrafollicular oxygenation. While inadequate vascularization is known to lead to hypoxia and atresia, current markers like AMH reflect the quantitative reserve but fail to capture the functional angiogenic capacity of the follicle. Therefore, identifying specific biomarkers of the vascular microenvironment remains a critical need for improving ART outcomes [6, 7].

Angiogenesis within the follicle is orchestrated by a complex network of factors, most notably Vascular Endothelial Growth Factor (VEGF) and angiopoietins [6]. While these markers have been extensively studied, there is a need to identify upstream regulators that modulate this vascular network under stress. One such candidate is SCUBE1 (Signal Peptide, CUB Domain, and EGF-Like Domain Containing 1), a cell-surface glycoprotein originally identified in vascular endothelial cells and platelets. Functionally, SCUBE1 acts as a critical co-receptor by forming complexes with ligands and receptors on the cell surface to amplify both VEGF and BMP signaling pathways. This amplification facilitates angiogenesis and supports cell survival under hypoxic conditions, providing a biological basis for its potential role in the follicular microenvironment [8, 9]. While direct evidence for SCUBE1 is emerging, its structural homology to SCUBE2 a known regulator of VEGFR2 signaling provides a compelling indirect biological basis for its role in the follicular vascular microenvironment. Although systemic levels of SCUBE1 are known to increase in ischemic pathologies such as acute coronary syndrome and ovarian torsion [10, 11], its specific role in physiological ovarian angiogenesis remains unexplored. Investigating SCUBE1 in the follicular fluid could provide novel insights into how the follicle adapts its vascular supply to match its rapid growth.

Given that follicular development is a hypoxia-sensitive and angiogenesis-dependent process, the hypothesis that SCUBE1 is present in follicular fluid and may play a regulatory role in ovarian physiology is highly plausible. However, no prior study has examined SCUBE1 specifically within the follicular microenvironment or its capacity to predict follicular performance.

To date, the presence of SCUBE1 in human follicular fluid and its relationship with ovarian response have not been investigated; this represents a significant gap in our understanding of the follicular molecular landscape. To the best of our knowledge, this is the first study to determine serum and follicular fluid SCUBE1 levels in infertile women and to prospectively examine the relationship of these levels with: (1) Ovarian reserve status (Poor, Normal, High), (2) Response to ovarian stimulation (number of retrieved oocytes), and (3) Clinical pregnancy outcomes. The primary hypothesis of this study is that SCUBE1, as a modulator of angiogenic signaling pathways, is present in human follicular fluid and its levels significantly differ according to the magnitude of the ovarian response. Specifically, we hypothesized that follicular fluid SCUBE1 levels would be higher in ‘High Responders’ reflecting an intensified angiogenic switch required to support a larger follicular cohort, thereby serving as a potential marker for the follicular vascular microenvironment rather than a direct indicator of oocyte genetic competence.

## Materials and methods

### Study design and ethical approval

This prospective cohort study was conducted between October 2021 and October 2022 at the Harran University Faculty of Medicine, Reproductive Medicine and IVF Center. The study protocol was designed in strict accordance with the ethical principles of the World Medical Association Declaration of Helsinki and was approved by the local Clinical Research Ethics Committee (Decision Date: 13.12.2021, Decision No: HRU/21.22.15). To minimize selection bias, eligible patients were recruited consecutively during the study period. Detailed information regarding the study’s purpose, procedures, and the use of biological samples was provided to all participants, and written informed consent was obtained.

### Study population

**Fig. 1:**
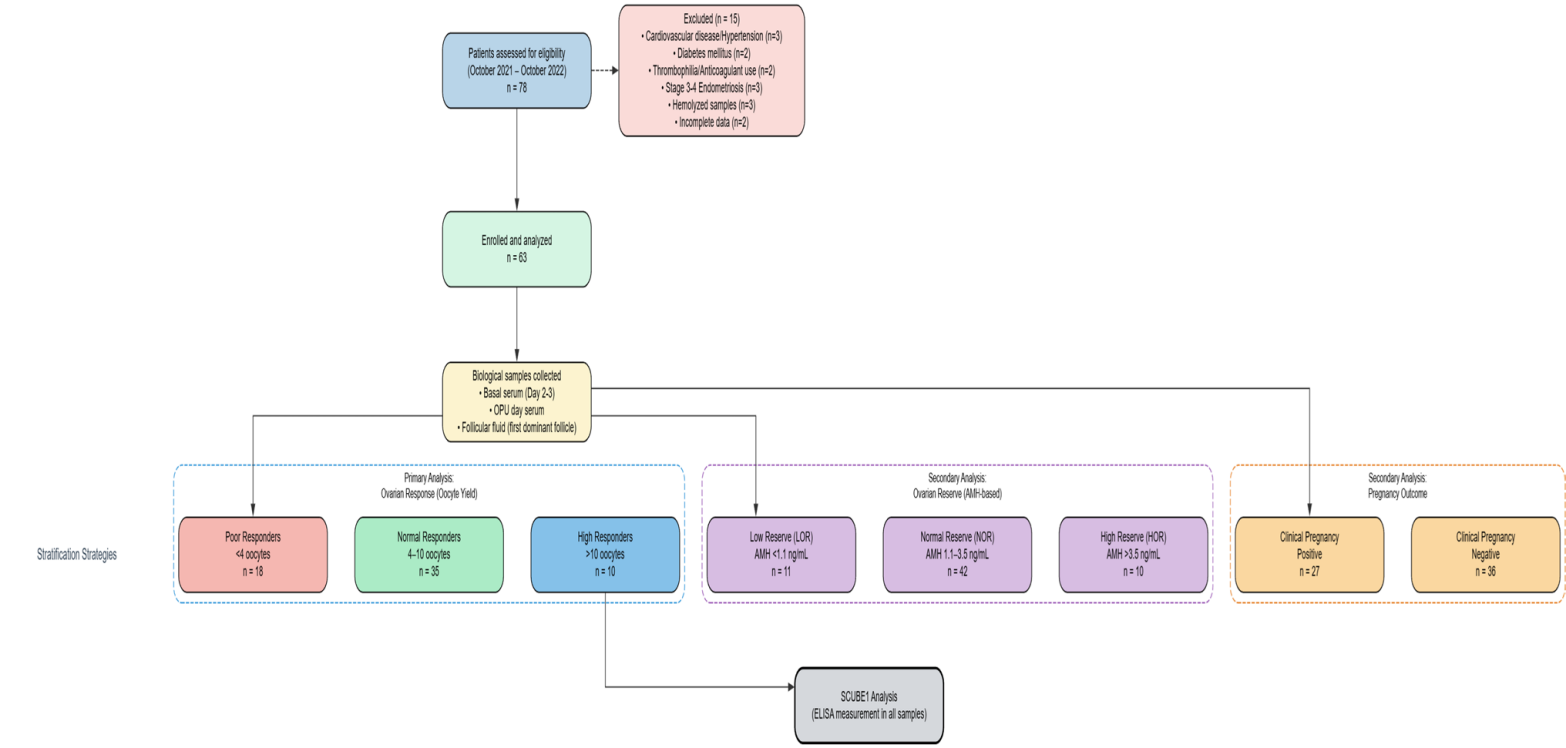
Study flow chart illustrating patient enrollment, exclusion criteria, sample collection process, and stratification according to ovarian response groups. A total of 78 patients were assessed for eligibility, of whom 15 were excluded based on predefined criteria. The remaining 63 patients underwent SCUBE1 analysis in paired serum and follicular fluid samples. CVD: Cardiovascular Disease; HTN: Hypertension; DM: Diabetes Mellitus; OPU: Oocyte Pick-Up

A total of 63 women aged 22–45 years who were admitted to the Intracytoplasmic Sperm Injection (ICSI) and Embryo Transfer (ET) program with a diagnosis of infertility were included in the study. The patient enrollment process, exclusion criteria, and group stratification are summarized in the study flow chart (Fig. 1). Polycystic Ovary Syndrome (PCOS) was diagnosed according to the Rotterdam criteria. The distribution of PCOS patients was balanced across the study groups.

### Power analysis

To detect a large effect size (Cohen’s *d* = 1.0) based on preliminary data of angiogenic markers in human follicular fluid, a sample size of at least 10 patients per response group was calculated as necessary with 80% power and a 5% type-1 error margin. By including a total of 63 patients (comprising 10 high and 18 poor responders), the study was adequately powered to evaluate the primary hypothesis regarding follicular fluid concentration differences across response categories, with a post hoc power exceeding 90%.

### Exclusion criteria

Given that SCUBE1 is a marker associated with vascular endothelial injury and thrombosis, systemic conditions that could influence serum levels were established as strict exclusion criteria. Accordingly, patients were excluded if they had: (1) a known history of cardiovascular disease, hypertension, or diabetes; (2) active infection or systemic inflammatory diseases (e.g., SLE, Rheumatoid Arthritis); (3) a history of thrombophilia or anticoagulant use; (4) uterine malformations (septum, bicornuate) or hydrosalpinx; and (5) a diagnosis of stage 3–4 endometriosis, (6) history of ovarian surgery or current smoking habit, which may alter vascular markers. Additionally, given the association of SCUBE1 with platelet activation, all samples were visually inspected for hemolysis. Visual inspection was utilized as a practical clinical standard to exclude gross hemolysis, and any pink-tinged samples were strictly discarded to prevent platelet-contamination bias. Although visual inspection is a common clinical practice, we acknowledge this as a limitation. Future studies should incorporate spectrophotometric assessment of hemoglobin to objectively rule out subtle micro-hemolysis.

### Study groups

To comprehensively evaluate the relationship of SCUBE1 levels with ovarian response capacity and reserve status, three distinct grouping strategies were applied:

1. **Ovarian Reserve Status:** Based on basal AMH levels; Low (LOR, < 1.1 ng/mL), Normal (NOR, 1.1–3.5 ng/mL), and High (HOR, > 3.5 ng/mL) reserve groups.
2. **Ovarian Response (Oocyte Yield):** Based on the total number of retrieved oocytes; Poor Responders (< 4 oocytes), Normal Responders (4–10 oocytes), and High Responders (> 10 oocytes).
3. **Pregnancy Outcome:** Groups were defined as clinical pregnancy positive or negative following embryo transfer.

### Controlled ovarian hyperstimulation (COH) protocol

A standard Gonadotropin-Releasing Hormone (GnRH) antagonist protocol was applied to all patients. Ovarian stimulation was initiated on day 2 or 3 of the menstrual cycle with recombinant Follicle Stimulating Hormone (rFSH) or human menopausal gonadotropin (hMG) injections. Starting doses (150–450 IU/day) were individualized according to the patient’s age, Body Mass Index (BMI), basal AMH, and Antral Follicle Count (AFC). Follicular development was monitored via serial transvaginal ultrasonography and serum estradiol (E2) measurements. When the leading follicle diameter reached 14 mm, a daily dose of 0.25 mg GnRH antagonist (cetrorelix acetate) was added to the regimen to prevent a premature Luteinizing Hormone (LH) surge. When at least three follicles reached a diameter of ≥ 17 mm, 250 mcg of recombinant human chorionic gonadotropin (rhCG) (Ovitrelle, Merck Serono) was administered to trigger final oocyte maturation.

### Oocyte retrieval, ICSI, embryo transfer, and luteal support

Oocyte retrieval (OPU) was performed under sedation and analgesia using transvaginal ultrasonography guidance 34–36 h after rhCG administration. The retrieved oocyte-cumulus complexes (OCC) were denuded using enzymatic (hyaluronidase) and mechanical methods. Microinjection (ICSI) was performed on mature oocytes at the Metaphase II (M2) stage. Fertilization was confirmed 16–18 h after the procedure by the presence of two pronuclei (2PN). Embryos were graded according to their morphological characteristics. Depending on the number and quality of available embryos, intrauterine transfer was performed under transabdominal ultrasound guidance on day 3 (cleavage stage) or day 5 (blastocyst stage) post-fertilization.

Luteal phase support was initiated on the evening of the OPU procedure (or the following day) and maintained using micronized vaginal progesterone (e.g., Progestan suppository 200 mg, 3 × 1) or progesterone vaginal gel (e.g., Crinone 8%, 1 × 1) until the pregnancy test day. In patients with a positive pregnancy test, luteal support was continued until the 10th–12th week of gestation.

### Collection and processing of biological samples

To dynamically monitor SCUBE1 levels, three different biological samples were obtained from each patient:

1. **Follicular phase serum (Basal):** Venous blood sample collected on the day stimulation commenced (Day 2–3 of the cycle).
2. **OPU day serum:** Venous blood sample collected on the morning of the oocyte retrieval procedure (36 h after hCG injection), immediately prior to the procedure.
3. **Follicular fluid (FF):** During the OPU procedure, the fluid from the first aspirated dominant follicle (diameter > 17 mm) was collected in a separate sterile tube. This ‘first follicle’ strategy was strictly applied to minimize the risk of blood contamination associated with subsequent punctures and to reduce biological variation ensuring the sample represents a mature follicle.

### Sample processing

Venous blood samples were centrifuged at 3000 rpm for 10 min to separate the serum. Follicular fluid samples were centrifuged at 600 × g for 5 min to remove cellular debris (granulosa cells and erythrocytes). The obtained serum and supernatant FF samples were stored frozen at − 80 °C until biochemical analysis.

### Outcome measures and definition of pregnancy

#### Primary outcome

The difference in follicular fluid SCUBE1 levels between different ovarian response groups (High vs. Poor Oocyte Response).

#### Secondary outcomes

1. Dynamic changes in serum SCUBE1 levels during the stimulation process (Basal vs. OPU Day).
2. Comparison of serum and follicular fluid SCUBE1 levels across different ovarian reserve groups (LOR, NOR, HOR).
3. The predictive value of SCUBE1 levels for clinical pregnancy.

### Pregnancy definition

Clinical pregnancy was defined as the visualization of an intrauterine gestational sac with a fetal heartbeat on transvaginal ultrasonography 4–6 weeks after embryo transfer.

### Measurement of SCUBE1 levels

SCUBE1 levels were measured using a commercial human signal peptide, CUB and EGF-like domain-containing protein 1 (SCUBE1) enzyme-linked immunosorbent assay (ELISA) kit (Bioassay Technology Laboratory (BT-LAB), Catalog No: E3142Hu, Shanghai, China). The assay sensitivity was 0.55 ng/mL, with a standard curve range of 1–400 ng/mL. Intra-assay and inter-assay coefficients of variation (CV) were < 8% and < 10%, respectively.

### Statistical analysis

Data were analyzed using the SPSS 24.0 (IBM Corp., Armonk, NY, USA) software package. Continuous variables were presented as Mean ± Standard Deviation (SD) or Median (Minimum–Maximum), while categorical variables were presented as frequencies and percentages (%). The conformity of variables to normal distribution was evaluated using the Shapiro–Wilk test and histograms. For comparisons between groups (e.g., High Response vs. Poor Response), the Mann–Whitney *U* test was used for non-normally distributed variables, and the Kruskal–Wallis test was used for comparisons involving more than two groups. The Jonckheere-Terpstra trend test was applied to evaluate the ordinal increasing trend across ovarian response groups. For pairwise comparisons between three groups (Poor vs Normal vs High), a post hoc analysis was performed. While Bonferroni correction was calculated (*p* < 0.017), *p*-values < 0.05 were considered nominally significant for exploratory biomarker analysis to avoid Type II errors in this pilot cohort. The Wilcoxon Signed Rank test (paired analysis) was used to compare measurements of the same patient at different time points (Basal Serum vs. OPU Serum) or in different compartments (Serum vs. FF). The relationship between SCUBE1 levels and oocyte numbers and embryo parameters was examined using Spearman correlation analysis. For regression and trend analyses, ovarian response was treated as an ordinal variable (Poor/Normal/High). For diagnostic accuracy (ROC) analysis, the variable was binarized as ‘High Responder’ (> 10 oocytes) versus ‘Non-High Responder’ (≤ 10 oocytes). ROC (Receiver Operating Characteristic) analysis was performed to determine the predictive power of follicular fluid SCUBE1 levels for high ovarian response. The optimal cut-off value was determined using the Youden Index (J = Sensitivity + Specificity − 1). SCUBE1 levels showed a non-normal distribution and were log-transformed for regression analysis; however, non-transformed (raw) data are presented in tables for clinical interpretability. Multicollinearity between independent variables was assessed using the Variance Inflation Factor (VIF), and no collinearity issues were detected (VIF < 2.5). Multivariate analyses were performed to determine the independent effects of clinically relevant variables on outcomes: Multiple Linear Regression analysis was used for factors affecting total oocyte count (Age, BMI, SCUBE1). For all analyses, a *p*-value of < 0.05 was considered statistically significant. Given the exploratory nature of this pilot biomarker study, p-values < 0.05 were considered nominally significant to identify potential trends, while Bonferroni corrections were calculated to maintain a conservative approach for primary comparisons.

## Results

### Demographic and basal clinical characteristics

As an exploratory pilot study, the following results identify patterns of SCUBE1 expression across clinical response categories. Of the 78 patients initially assessed for eligibility, 15 were excluded according to predefined criteria (Fig. 1). A total of 63 infertile women undergoing IVF/ICSI treatment were included in the final analysis. The mean age of the study population was 30.76 ± 5.07 years (range: 22–45), and the mean Body Mass Index (BMI) was 25.19 ± 4.06 kg/m^2^. The basal hormonal profiles and ovarian reserve parameters of the patients are detailed in Table 1. The mean basal FSH level was 7.64 ± 3.41 mIU/mL, the mean AMH level was 2.07 ± 1.13 ng/mL, and the mean Antral Follicle Count (AFC) was 9.17 ± 4.73. As a result of ovarian stimulation, an average of 6.43 ± 4.03 oocytes were retrieved per patient, and an average of 4.14 ± 2.96 of these were evaluated as mature oocytes at the Metaphase II (M2) stage.

**Table 1:** Demographic, hormonal, and clinical characteristics of the study population (*N* = 63)

Parameter	Mean ± SD/*n* (%)	Median (Min–Max)
Age (years)	30.76 ± 5.07	30.0 (22.0–45.0)
BMI (kg/m^2^)	25.19 ± 4.06	24.6 (17.0–38.0)
Duration of ınfertility (years)	4.8 ± 2.6	4.0 (1.0–11.0)
Infertility etiology		
Male factor	20 (31.7%)	-
Tubal factor	8 (12.7%)	-
Unexplained	18 (28.6%)	-
PCOS	12 (19.0%)	-
Mixed/other	5 (7.9%)	-
Type of ınfertility		
Primary	45 (71.4%)	-
Secondary	18 (28.6%)	-
Basal FSH (mIU/mL)	7.64 ± 3.41	7.05 (0.93–25.74)
Basal LH (mIU/mL)	4.33 ± 2.19	4.17 (0.10–12.98)
Basal estradiol (pg/mL)	38.64 ± 15.11	42.66 (11.71–79.65)
AMH (ng/mL)	2.07 ± 1.13	1.85 (0.03–4.69)
Antral follicle count (AFC)	9.17 ± 4.73	9.0 (2–22)
Duration of stimulation (days)	10.2 ± 1.7	10.0 (8.0–14.0)
Total gonadotropin dose (IU)	2436.2 ± 990.5	2250.0 (1125–5400)
Estradiol on hCG day (pg/mL)	1452.4 ± 845.1	1285.0 (310–4250)
Total retrieved oocytes	6.43 ± 4.03	6.0 (1–22)
M2 Oocytes	4.14 ± 2.96	4.0 (0–15)
Transfer day (Day 3/Day 5)	40/23	-
Number of embryos transferred	1.2 ± 0.4	1 (1–2)

*SD* standard deviation, *BMI* body mass index, *FSH* follicle stimulating hormone, *LH* luteinizing hormone, *AMH* anti-müllerian hormone

### SCUBE1 dynamics during ovarian stimulation

The variation in SCUBE1 levels throughout the stimulation period was analyzed using paired samples obtained from the same patients. A statistically significant and marked decline in *systemic* SCUBE1 levels was observed in conjunction with gonadotropin stimulation (*p* < 0.001). The mean serum SCUBE1 level, which was 159.77 ng/mL at the onset of the follicular phase (Day 3), decreased to 115.67 ng/mL on the day of OPU. Conversely, when the entire cohort was considered, no statistically significant difference was found between OPU-day serum and FF SCUBE1 levels (*p* = 0.441), suggesting an overall equilibrium (Table 2). However, the compartment-specific elevation observed specifically in high responders (Table 3) points to a localized phenomenon within the hyperactive ovary.

**Table 2:** Comparison of serum and follicular fluid SCUBE1 levels

Sampling time and type	SCUBE1 level (ng/mL) (Mean ± SD)	Change	*P*-value
1. Follicular phase serum (Day 3)	159.77 ± 122.82	Ref	-
2. OPU day serum	115.67 ± 122.16	↓ Decrease	< 0.001
3. Follicular fluid (FF)	101.99 ± 96.81	(vs. Serum 2)	0.441

(Wilcoxon Signed Rank Test. The difference between 1 and 2 is significant; the difference between 2 and 3 is not significant.)

**Table 3:** Comparison of SCUBE1 levels according to Ovarian Response Groups

Variable	Poor response (< 4 oocytes) (*n* = 18)	Normal response (4–10 oocytes) (*n* = 35)	High response (> 10 oocytes) (*n* = 10)	*P*-value*
Age (years)	32.50 ± 5.43	30.54 ± 5.01	28.40 ± 3.92	0.084
AMH (ng/mL)	1.03 ± 0.98	2.58 ± 1.57	5.33 ± 2.40	< 0.001
Follicular phase serum SCUBE1	152.20 ± 110.50	149.23 ± 122.05	210.23 ± 145.30	0.412
OPU day serum SCUBE1	99.19 ± 85.40	108.74 ± 126.62	169.45 ± 130.20	0.254
Follicular fluid (FF) SCUBE1	84.66 ± 73.15	90.30 ± 88.77	174.04 ± 112.45	0.043

Data presented as Mean ± SD. Statistical Analysis: The overall difference between groups was evaluated using the Kruskal–Wallis test (*p* = 0.043). Pairwise post hoc analysis (Mann–Whitney *U*) revealed a significant difference specifically between High and Poor responder groups. Furthermore, the Jonckheere-Terpstra trend test confirmed a significant increasing trend in Follicular Fluid SCUBE1 levels across response groups (Standardized J-T Statistic = 2.145, *p* = 0.032)

### SCUBE1 levels according to ovarian response

**Fig. 2:**
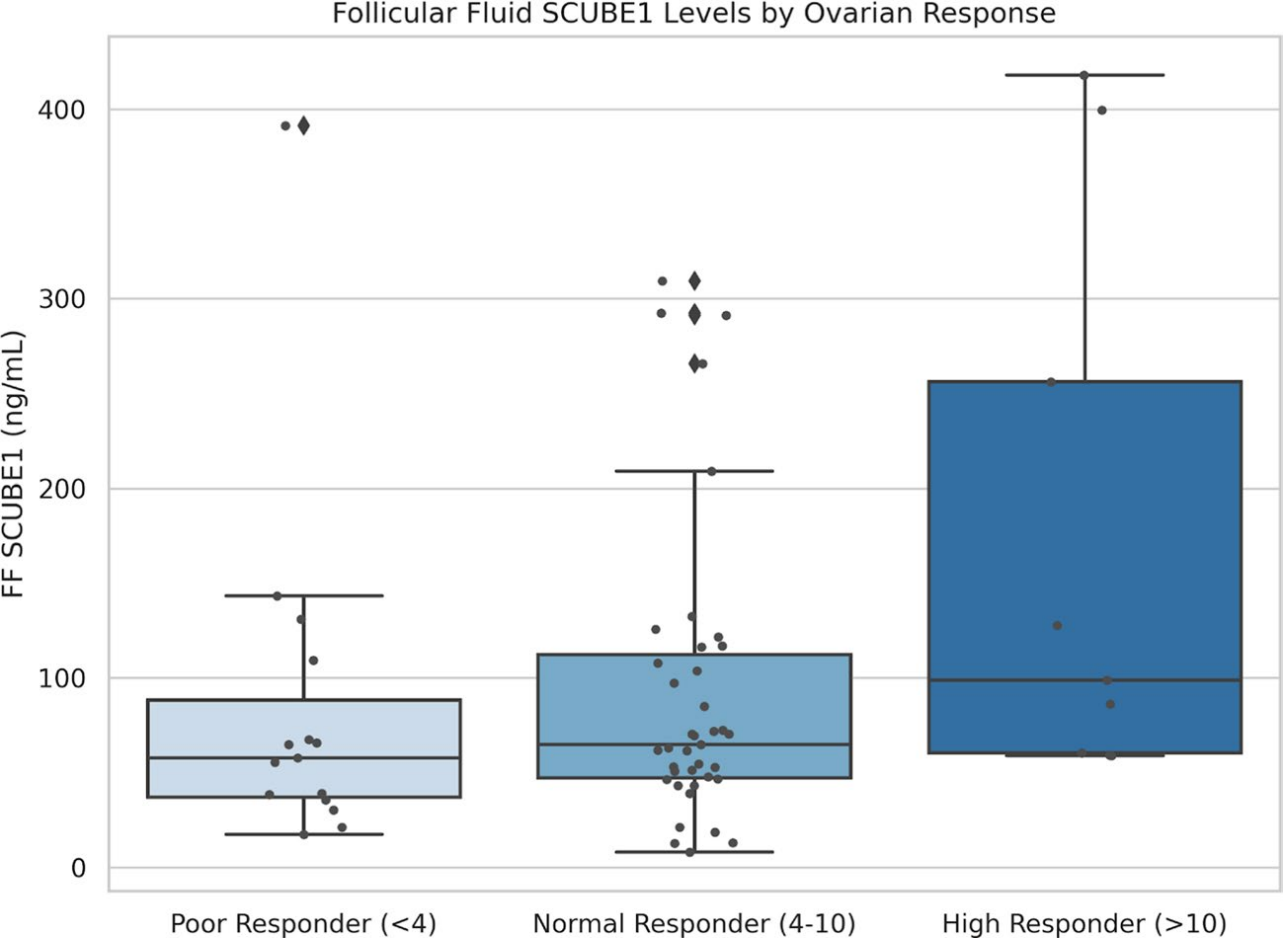
Box-plot graph showing Follicular Fluid SCUBE1 levels across Ovarian Response groups. The ‘High Responder’ group (> 10 oocytes) exhibits significantly higher levels compared to ‘Poor Responders’ (< 4 oocytes) (*p* = 0.043, Kruskal–Wallis with post hoc Mann–Whitney *U*). Outliers are indicated by dots. Horizontal lines represent medians; boxes represent interquartile ranges

Upon examining the relationship between SCUBE1 and “Ovarian Response,” the primary endpoint of the study, a distinct divergence was observed within the follicular microenvironment. When patients were stratified according to the number of retrieved oocytes into Poor Responders (< 4 oocytes), Normal Responders (4–10 oocytes), and High Responders (> 10 oocytes), systemic (serum) SCUBE1 levels remained comparable across groups; however, follicular fluid SCUBE1 levels exhibited a marked increase in the “High Responder” group (Table 3) (Fig. 2). Notably, the FF SCUBE1 level in the High Responder group (174.04 ng/mL) was approximately twofold higher than that of the Poor Responder group (84.66 ng/mL), a difference that was statistically significant (*p* = 0.043).

### Relationship between SCUBE1 and oocyte parameters

No statistically significant relationship was detected between follicular fluid SCUBE1 levels and the oocyte maturation rate (M2/Total Oocytes; *r* = 0.101, *p* > 0.05) or fertilization rate (2PN/M2; *r* = 0.010, *p* > 0.05) (Table 5). Similarly, no linear relationship was observed in the Spearman correlation analysis regarding the total oocyte count (*r* = 0.149, *p* = 0.244). However, when the behavior of SCUBE1 levels across categorical response groups was examined, a distinct pattern emerged. When the ordinal increasing trend among groups was evaluated using the Jonckheere-Terpstra Trend Test, a statistically significant upward trend in FF SCUBE1 levels was detected as the oocyte yield groups increased (Standardized J-T Statistic = 2.145, *p* = 0.032, indicating a positive trend). This finding supports the hypothesis that the relationship between SCUBE1 and oocyte number exhibits a “threshold effect” becoming pronounced only after exceeding a certain follicular capacity rather than a simple linear function.

### Diagnostic evaluation (ROC analysis)

**Fig. 3:**
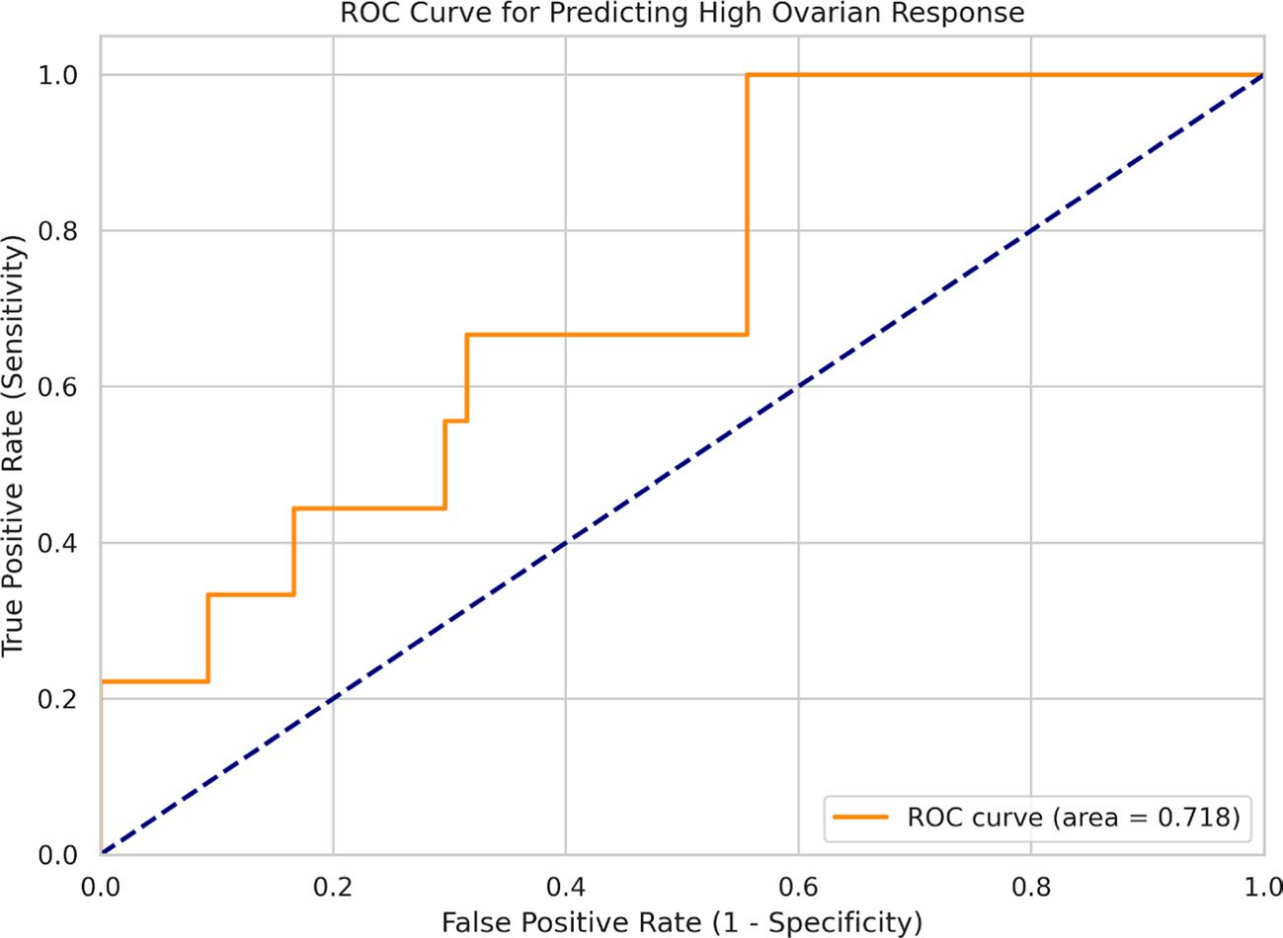
ROC curve analysis for Follicular Fluid SCUBE1 in predicting High Ovarian Response (> 10 oocytes). AUC = 0.718 (95% CI: 0.532–0.904)

The predictive power of Follicular Fluid SCUBE1 levels for “High Responders” (> 10 retrieved oocytes) was evaluated using ROC analysis. The AUC value was determined to be 0.718 (95% CI: 0.532–0.904, *p* = 0.048) (Fig. 3). This value indicates that the diagnostic accuracy of SCUBE1 is ‘fair.’ At the determined cut-off value of 59.13 ng/mL, sensitivity was calculated as 100%, specificity as 44.4%, Positive Predictive Value (PPV) as 25%, and Negative Predictive Value (NPV) as 100%. The high NPV (100%) is clinically valuable as it indicates that the probability of achieving a ‘High Response’ (high oocyte yield) is extremely low in patients below this threshold value.

### Relationship with ovarian reserve and pregnancy outcomes

SCUBE1 levels were also analyzed in terms of AMH-based ovarian reserve classification (LOR, NOR, HOR) and Clinical Pregnancy outcomes. Unlike ovarian response (oocyte count), neither reserve status nor pregnancy success was found to be associated with SCUBE1 levels (Table 4). Furthermore, no correlation was detected between SCUBE1 and oocyte maturation rate (M2/Total) or fertilization rate (*r* = 0.10 and *r* = 0.01, *p* > 0.05, respectively).

**Table 4:** SCUBE1 levels according to pregnancy status and ovarian reserve

Groups	*n*	Follicular phase serum SCUBE1 (ng/mL)	OPU day serum SCUBE1 (ng/mL)	Follicular fluid SCUBE1 (ng/mL)	*P*-value
Pregnancy outcome					
Non-pregnant	36	156.50 ± 126.07	101.20 ± 100.64	102.57 ± 93.38	0.396
Pregnant	27	164.13 ± 120.57	134.96 ± 145.90	101.21 ± 103.14	
Ovarian reserve					
Low (LOR)	11	165.14 ± 93.98	148.00 ± 134.82	108.87 ± 91.36	0.881
Normal (NOR)	42	154.89 ± 127.52	100.32 ± 108.12	102.91 ± 99.88	
High (HOR)	10	167.87 ± 139.45	129.83 ± 138.13	94.68 ± 89.84	

*P*-values refer to the comparison of Follicular Fluid SCUBE1 levels between the groups (Mann–Whitney *U *test for pregnancy outcome; Kruskal–Wallis test for Ovarian Reserve). No significant differences were observed in serum levels

### Correlation analysis

The following table demonstrates the correlation of serum and FF SCUBE1 levels with clinical parameters (Table 5).

**Table 5:** Correlation matrix between SCUBE1 levels and clinical parameters (Spearman)

Parameter	FF SCUBE1 (*r*)	*P*-value	Serum SCUBE1 (*r*)	*P*-value
Total Oocytes	0.149	0.244	0.110	0.395
M2 Oocytes	0.203	0.110	0.095	0.460
Maturation rate (M2/Total)	0.101	> 0.05	-	-
Fertilization rate (2PN/M2)	0.010	> 0.05	-	-
AMH	0.089	0.488	0.054	0.672
Age	− 0.150	0.240	− 0.112	0.380
BMI	− 0.098	0.445	− 0.085	0.510

Although there is a trend towards a weak positive relationship (*r* = 0.20) between FF SCUBE1 and M2 oocytes, it is not significant. This supports the observation that SCUBE1 exhibits a “threshold effect” rather than a linear relationship; specifically, it peaks only in the “High Responder” group

### Multivariate analysis

A multiple linear regression analysis was conducted to evaluate the independent effects of Age, BMI, and Follicular fluid (FF) SCUBE1 levels on the total oocyte count (Table 6). AMH was not included in the multivariate regression model due to its strong collinearity with the dependent variable (oocyte count), as AMH is inherently a strong predictor of ovarian response. The regression model was statistically significant (F(3,59) = 4.47, *p* = 0.007) and explained approximately 18.5% of the variance in oocyte yield (*R*^2^ = 0.185). According to the analysis, Age was identified as a significant independent predictor, negatively affecting the oocyte count (*β* =  − 0.285, *p* = 0.033). Specifically, for every one-year increase in age, the total number of retrieved oocytes decreased by approximately 0.22 units when other variables were held constant. In contrast, FF SCUBE1 levels showed a positive trend with oocyte yield (*β* = 0.218, *p* = 0.087), which did not reach independent statistical significance but suggests a relevant clinical signal in this pilot cohort. While age remains the primary independent predictor of response, this observed trend in a pilot cohort suggests that SCUBE1 may play a supportive role in the follicular vascular microenvironment that warrants further validation in larger, more powered studies. Body Mass Index (BMI) did not show a significant effect on the outcome (*p* = 0.496).

**Table 6:** Multiple linear regression analysis evaluating factors associated with total oocyte yield

Independent variables	Unstandardized coefficients (B)	Std. Error (SE)	Standardized coefficients (β)	*t*	*P*-value	95% Confidence interval for B
(Constant)	18.450	5.210	—	3.541		8.025–28.875
Age (years)	− 0.224	0.103	− 0.285	−2.182	0.033*	− 0.430 – −0.018
BMI (kg/m^2^)	− 0.085	0.124	− 0.088	−0.685	0.496	− 0.334–0.164
FF SCUBE1 (ng/mL)	0.009	0.005	0.218	1.739	0.087**	− 0.001–0.019

Dependent Variable: Total Number of Retrieved Oocytes. Model Summary: *R*^2^ = 0.185; Adjusted *R*^2^ = 0.144; ANOVA *p* = 0.007. *B* unstandardized regression coefficient, *SE* standard error, *(β)* standardized regression coefficient, *CI* confidence interval, *FF* follicular fluid. *Indicates statistical significance (*p* < 0.05). **Indicates borderline significance/trend (0.05 < *p* < 0.10)

## Discussion

This study represents the first investigation to examine the relationship between follicular fluid SCUBE1 levels and the response to controlled ovarian stimulation in infertile women. The most important finding of our study was the specific elevation of follicular fluid SCUBE1 concentrations in the high responder group, suggesting a potential role in supporting the vascular demands of a large follicular cohort. It has been previously reported in the literature that classic angiogenic factors, such as VEGF and PlGF, exhibit a positive correlation with follicular development [12, 13]. SCUBE1 is known to modulate several growth factors, particularly through its C-terminal CUB and EGF-like domains (8). Although, we did not measure VEGF and PlGF in FF of patients, our findings demonstrated that follicular SCUBE1 levels in high follicular activity may suggest that it is an integral component of the angiogenic system and accumulates intensely within the follicular microenvironment. A potential limitation of our study is the absence of simultaneous VEGF measurements in the follicular fluid. While SCUBE1 is known to act as a co-receptor that modulates VEGF signaling, our study focused on identifying SCUBE1 as a novel, independent element of the follicular microenvironment. Although direct correlation with VEGF was not assessed in this pilot cohort, the significant elevation of SCUBE1 in high responders a group known for high VEGF activity strongly supports the role of SCUBE1 in the recruitment and stabilization of the follicular vascular network [14–17]. Future studies integrating both markers are needed to clarify the synergistic dynamics between SCUBE1 and the VEGF/VEGFR2 axis during human folliculogenesis. Therefore, future studies should measure SCUBE1 alongside VEGF and its soluble receptor (sVEGFR2) in paired FF samples to elucidate their interplay and relative contributions to follicular angiogenesis.

**Fig. 4:**
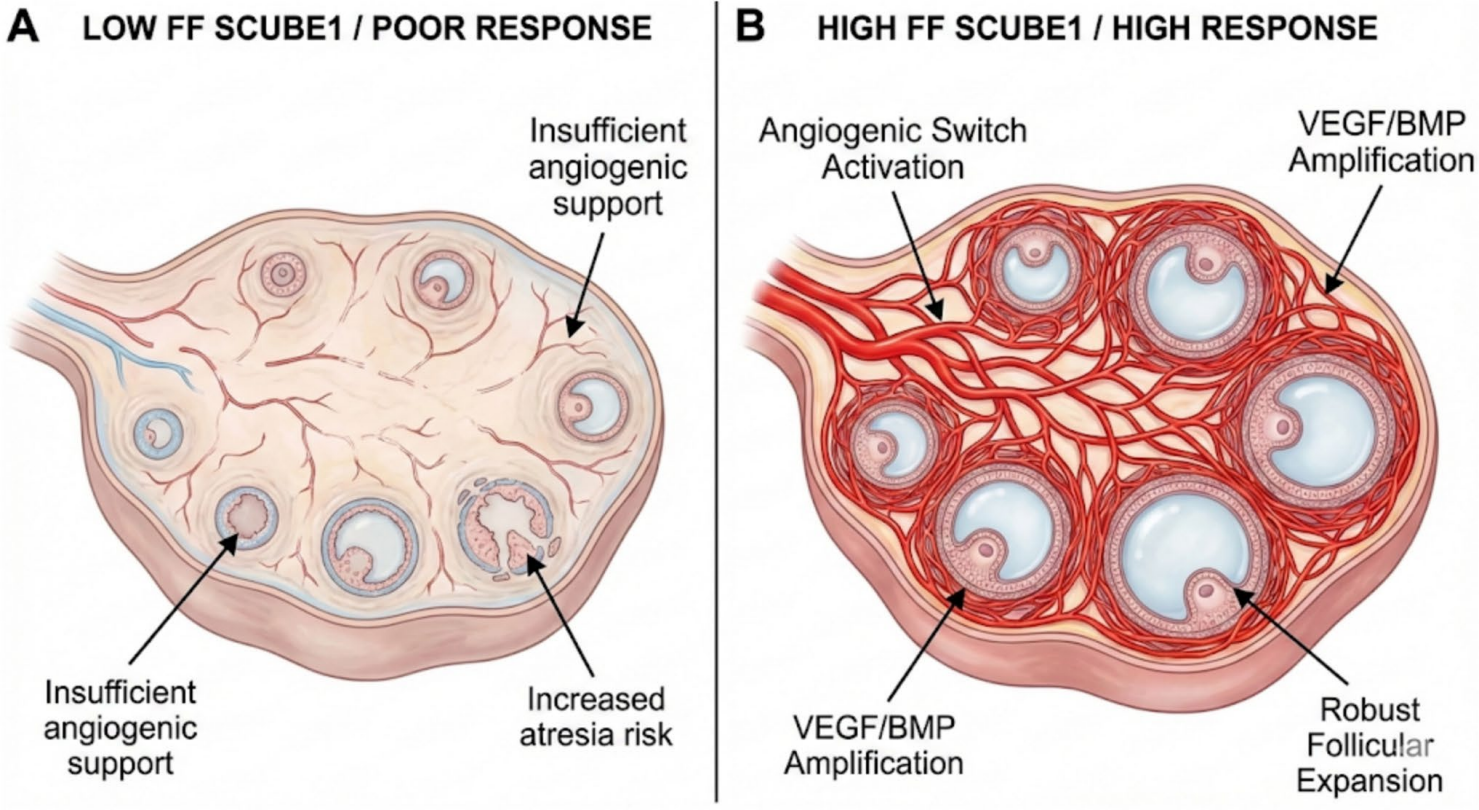
Proposed role of SCUBE1 in the follicular angiogenic switch. (**A**) In poor ovarian response, low follicular fluid (FF) SCUBE1 levels are associated with attenuated VEGF/BMP signaling, resulting in inadequate thecal vascularization, limited nutrient/gonadotropin delivery, and increased risk of follicular atresia. (**B**) In high ovarian response, elevated FF SCUBE1 levels potentiate VEGF/VEGFR2 and BMP signaling pathways. This amplification triggers a robust ‘angiogenic switch,’ leading to dense thecal vessel formation, optimal follicular perfusion, and the synchronized development of a large, healthy follicular cohort

Oocyte maturation occurs within the FF, where metabolic exchange between granulosa cells and the oocyte takes place. This dynamic microenvironment is rich in steroid hormones, growth factors, cytokines, and various metabolites. Folliculogenesis is one of the most rapid tissue proliferation and remodeling events in the human body, and this rapid growth must be supported by intense angiogenesis and increased vascular permeability. Establishing a rich vascular network within the theca layer is vital to meet the increasing metabolic demands of the developing follicle, ensure gonadotropin delivery to target cells, and maintain intrafollicular oxygenation. It is well established that inadequate vascularization leads to follicular hypoxia, atresia, and compromised oocyte quality [6, 18, 19]. Therefore, molecules associated with angiogenesis and vascular endothelial function (e.g., VEGF, HIF-1α) may serve as potential indicators of follicular health and, indirectly, embryo developmental potential. Given the structure of SCUBE1, which contains EGF-like repeats and interacts with adhesion molecules (such as PEAR1) on the endothelial cell surface, the high levels we detected may reflect the stabilization of the follicular vascular network. Our results indicate a 'threshold effect' rather than a linear correlation; SCUBE1 levels do not rise incrementally with each oocyte but surge significantly only when the follicular cohort exceeds a certain capacity (high response), suggesting an 'angiogenic switch' mechanism. The increase observed in high responders can be interpreted as an adaptive vascular response developed by the growing multi-follicular cohort against hypoxia. Indeed, it is known that follicular atresia accelerates when angiogenic support is insufficient [6, 20]; the low SCUBE1 levels in our poor responder group support this vascular insufficiency hypothesis. The specific elevation in high responders FF suggests that SCUBE1 levels may be a key component of the angiogenic switch the critical transition from a quiescent to a highly vascularized state required for the development of multiple dominant follicles. Without this vascular surge, marked by high SCUBE1, the cohort may fail to progress (Fig. 4).

The physiological basis for the high SCUBE1 levels observed in the high responder group in our study can be explained by the molecule’s regulatory roles in paracrine signaling, in addition to its vascular functions. The ovary is one of the rare organs where dynamic and continuous tissue remodeling occurs throughout folliculogenesis, and angiogenesis is cyclically repeated [7]. Lin et al. (2023) demonstrated through comprehensive molecular analyses that SCUBE1 functions as a ‘co-receptor’ by forming complexes with Bone Morphogenetic Protein (BMP) ligands and receptors on the cell surface, thereby amplifying BMP signaling [8]. Given that factors such as BMP15 and GDF9 are critical for granulosa cell proliferation during follicular development, the elevated SCUBE1 levels in follicular fluid may support follicular survival by potentiating these growth signals. Furthermore, SCUBE1 has been shown to reduce apoptosis and promote cellular repair in epithelial cells (e.g., renal tubular cells) under ischemic stress [8]. In light of these data, the increased SCUBE1 levels in high responders likely reflect the presence of a healthy granulosa cell pool that is resistant to apoptosis, possesses strong BMP signaling, and exhibits high proliferative capacity within the follicular microenvironment. Our findings are also consistent with the quantitative proteomic analysis by Ambekar et al. (2015), which identified SCUBE1 downregulation in the follicular fluid of women with PCOS [21]. Given that PCOS is characterized by arrested follicular development, the lower levels observed in their study and the similarly lower concentrations in our poor responder group further suggest that a critical threshold of SCUBE1 is necessary for successful follicular maturation and basement membrane remodeling. This supports our hypothesis that SCUBE1 serves as a functional marker of follicular competence, where its insufficiency may lead to stalled folliculogenesis.

Another notable finding of our study is that systemic (serum) SCUBE1 levels showed a significant decrease (*p* < 0.001) throughout the controlled ovarian hyperstimulation process, while intrafollicular levels remained elevated. This paradoxical decline in systemic SCUBE1 can be explained by several complementary mechanisms. First, the rapidly developing multi-follicular cohort creates a substantially expanded endothelial surface area within the ovarian vasculature, which may actively sequester circulating SCUBE1 from the systemic pool. Given that SCUBE1 binds to endothelial cells and functions as a co-receptor in VEGF and BMP signaling pathways [8], its recruitment to sites of active angiogenesis is biologically plausible. Second, this pattern may reflect increased local consumption of SCUBE1 at the ovarian level, where intense vascular remodeling and neoangiogenesis occur during follicular development. Similar compartment-specific dynamics have been described for other angiogenic factors such as PlGF, which shows differential regulation in follicular fluid versus serum during ovarian stimulation [12]. Third, the contrasting dynamics systemic decrease versus sustained high intrafollicular levels strongly support the hypothesis of local SCUBE1 production by ovarian cells (granulosa or theca cells), creating a follicle-specific angiogenic microenvironment independent of systemic delivery. This concept aligns with the established role of localized angiogenic mediators in ensuring adequate vascularization of the metabolically demanding developing follicle [6, 18]. Future studies incorporating paired measurements of other endothelial markers and assessing SCUBE1 mRNA expression in granulosa cells would help clarify whether the elevated intrafollicular SCUBE1 originates from local synthesis or selective uptake from circulation.

While our initial focus included oocyte quality, it is important to note that clinical pregnancy and live birth rates are influenced by multifactorial elements, including embryo euploidy and endometrial receptivity, which may be independent of the initial follicular angiogenic surge. The lack of association between SCUBE1 and fertilization rates or clinical pregnancy in our study suggests that SCUBE1 is more reflective of the 'quantitative' vascular support system (fuel supply) rather than the 'qualitative' genetic competence of the oocyte. Furthermore, the absence of live birth data the ultimate gold standard in ART is a limitation; however, our findings primarily highlight SCUBE1 as a functional marker of the follicular microenvironment's response to stimulation.

One of the most clinically relevant outputs of our study is the ROC analysis evaluating the diagnostic performance of FF SCUBE1 levels. The obtained AUC value of 0.718 indicates a fair diagnostic performance, primarily driven by a positive trend observed in our multivariate model. Although AMH and AFC are currently accepted as gold standards for ovarian reserve assessment and generally show higher diagnostic accuracy (> 0.80 AUC) [22], these markers offer limited information regarding the internal (intrafollicular) physiology of the follicle. The 100% Negative Predictive Value (NPV) detected at our determined cut-off value of 59.13 ng/mL is clinically highly valuable. This finding indicates that in a patient with FF SCUBE1 levels below this threshold, the probability of achieving a high response (high oocyte yield) is virtually impossible. Although the specificity (44.4%) at the determined cut-off is relatively low, limiting its use as a standalone diagnostic tool, the high sensitivity makes it valuable for ruling out poor vascular support. It is important to note that while the specificity of SCUBE1 is modest, its 100% Negative Predictive Value (NPV) at the 59.13 ng/mL threshold is its most clinically relevant feature. This indicates that SCUBE1 may function effectively as a 'rule-out' marker; patients falling below this threshold are highly unlikely to achieve a high oocyte yield, potentially signaling a vascular insufficiency in the follicular cohort. As stated by Jankowska-Ziemak et al. (2025), ovarian angiogenesis is a prerequisite for follicular growth [7]; thus, low SCUBE1 can be considered an indicator of an inadequate vascular network and suppressed follicular support. In this context, SCUBE1 can be used as a complementary quality control marker, particularly in the management of patients with an unexpected poor response (hypo-responders), to understand whether the issue stems from follicular vascularization and to plan future cycles accordingly. From a clinical perspective, a low FF SCUBE1 level (< 59.13 ng/mL) could be used to confidently rule out a high ovarian response. Identifying such patients below this threshold may help clinicians tailor stimulation strategies or consider adjuvant therapies aimed at improving follicular perfusion.

Another important finding shedding light on clinical practice is the lack of difference in FF SCUBE1 concentrations between the Low Ovarian Reserve (LOR) and Normal Ovarian Reserve (NOR) groups. Contrary to expectations, a reduction in reserve did not lead to a decrease in this angiogenic factor within the follicular fluid. This phenomenon suggests a compensatory vascular mechanism within the dominant follicle. Although the total ovarian reserve decreases, the dominant follicle selected for ovulation maintains its micro-environmental quality and angiogenic support to ensure oocyte competence. SCUBE1 indicates how well the existing egg is nourished (micro-environmental quality) rather than how many eggs remain (reserve). This discrepancy between High Reserve’ (AMH-based) and High Responder (Oocyte-based) groups regarding SCUBE1 levels is particularly noteworthy. While the High Reserve group represents the potential ovarian pool, the High Responder group represents the actual recruited cohort undergoing active metabolism. The fact that SCUBE1 is elevated only in the High Responder group but not in the High Reserve group supports the hypothesis that SCUBE1 is not a static marker of the resting pool (like AMH), but rather a dynamic mediator of active angiogenesis triggered during the rapid growth of multiple follicles. Additionally, Table 3 groups are defined by functional outcome (oocyte yield), whereas Table 4 groups rely on hormonal biomarkers; thus, a lack of complete overlap is expected. This reinforces the concept that SCUBE1 is a marker of dynamic follicular activity rather than static reserve, potentially explaining the clinical discordance sometimes observed between normal AMH levels and a poor ovarian response.

From a clinical standpoint, our findings suggest that FF SCUBE1 measurement could be incorporated into a multi-marker panel for predicting ovarian response. Specifically, in patients who respond poorly despite adequate AMH levels (‘unexpected poor responders’), low FF SCUBE1 might indicate compromised follicular vascularization rather than diminished ovarian reserve. This distinction could guide clinicians in tailoring subsequent treatment cycles for instance, by considering adjuvant therapies aimed at improving follicular blood flow. However, prospective validation studies with predefined cut-offs are essential before clinical implementation.

Our study has several limitations that should be acknowledged. First, while the ‘first-follicle’ sampling strategy minimized blood contamination risk, it may not capture the metabolic heterogeneity of the entire follicular cohort, especially in high responders. Second, although we performed strict visual inspection to exclude hemolyzed samples, future studies should employ spectrophotometric assessment of hemoglobin or platelet-specific markers (e.g., PF4) to objectively rule out subtle, platelet-derived SCUBE1 contamination. Third, while PCOS patients were distributed across groups, the absence of a dedicated subgroup analysis limits our understanding of SCUBE1 dynamics within this specific pathophysiology. Fourth, the exploratory nature of this pilot study led us to consider *p*-values < 0.05 as nominally significant to avoid Type II errors, with the understanding that Bonferroni correction yields a more stringent threshold (*p* < 0.017). Consequently, the significant finding for FF SCUBE1 (*p* = 0.043) should be interpreted as strong preliminary evidence. Fifth, the relatively small sample size in the high responder group (*n* = 10) likely contributed to the borderline significance (*p* = 0.087) of SCUBE1 in the multivariate model, underscoring the need for validation in larger cohorts. Finally, the wide age range (22–45 years), while reflective of a real-world ART population, may introduce confounding; future age-stratified analyses are warranted. Despite these limitations, this pilot study provides the first clinical evidence of SCUBE1 in the human follicular microenvironment and establishes a crucial foundation for such future research.

## Conclusion

This pilot study provides the first evidence that SCUBE1, a glycoprotein involved in angiogenesis, is present in human follicular fluid and serves as a dynamic biomarker of the follicular vascular microenvironment. We demonstrate that follicular fluid SCUBE1 levels are significantly elevated in high ovarian responders, reflecting an intensified “angiogenic switch” critical for supporting the development of a large follicular cohort during controlled stimulation. The robust negative predictive value of a low SCUBE1 level offers a clinically useful “rule-out” tool for identifying patients with probable vascular insufficiency, even in the presence of an adequate quantitative ovarian reserve. Crucially, SCUBE1 is not a marker of oocyte genetic competence or pregnancy potential but rather a specific indicator of follicular vascular performance. These findings position SCUBE1 as a novel, complementary biomarker that could help explain and manage cases of unexpected poor ovarian response. Future large-scale, multi-center studies are warranted to validate the proposed diagnostic threshold and to investigate the potential of modulating this pathway to optimize follicular vascular support in personalized ART protocols.

## Data Availability

The datasets generated and/or analyzed during the current study are available from the corresponding author on reasonable request.
